# Supplementary figures and images for: Neurorobotics Workshop for High School Students Promotes Competence and Confidence in Computational Neuroscience
Source: Front Neurorobot. 2020 Feb 13;14:6. doi: 10.3389/fnbot.2020.00006 (PMC7033397; doi:10.3389/fnbot.2020.00006)

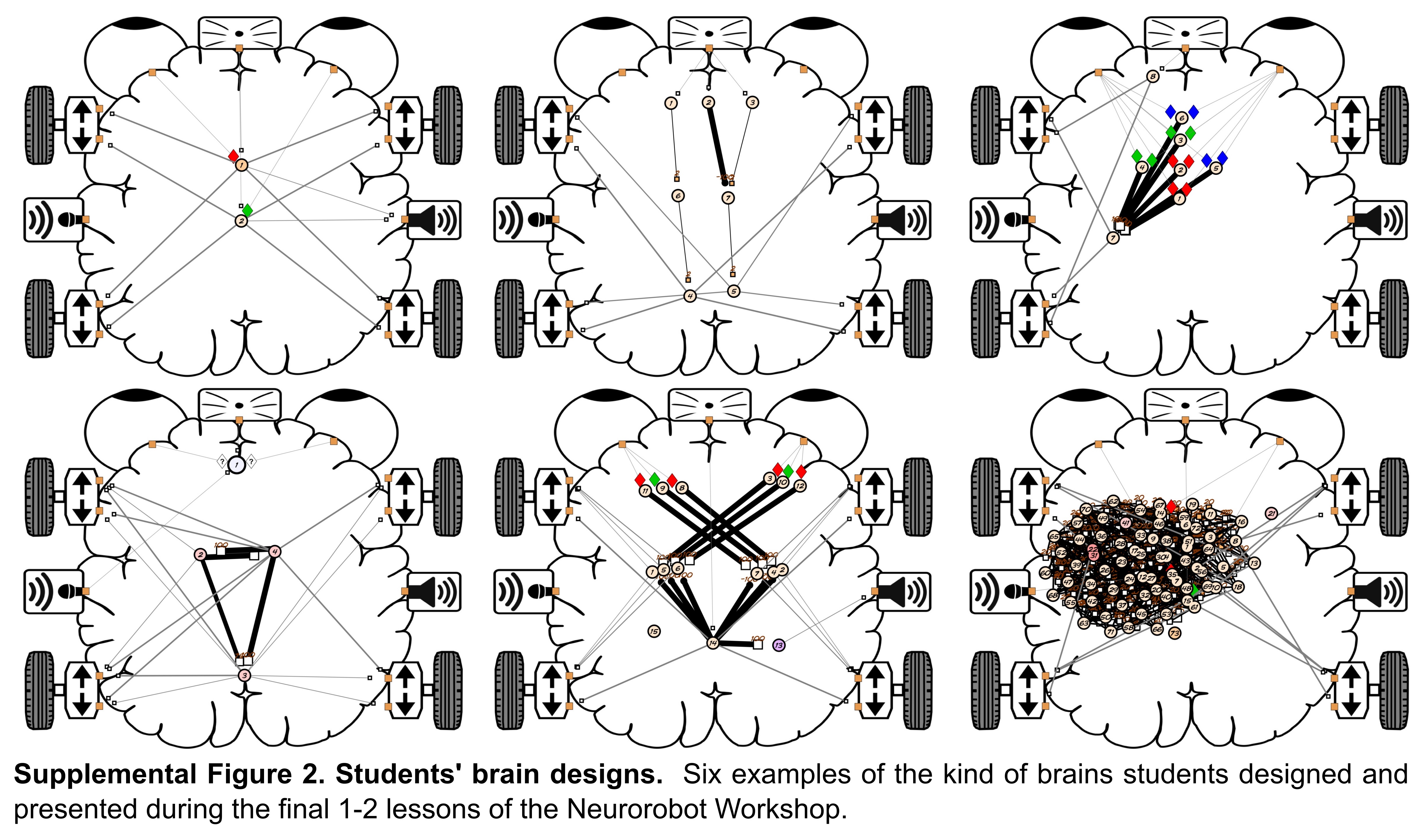

Supplement: Supplementary file 1 [file Image_2.JPEG]

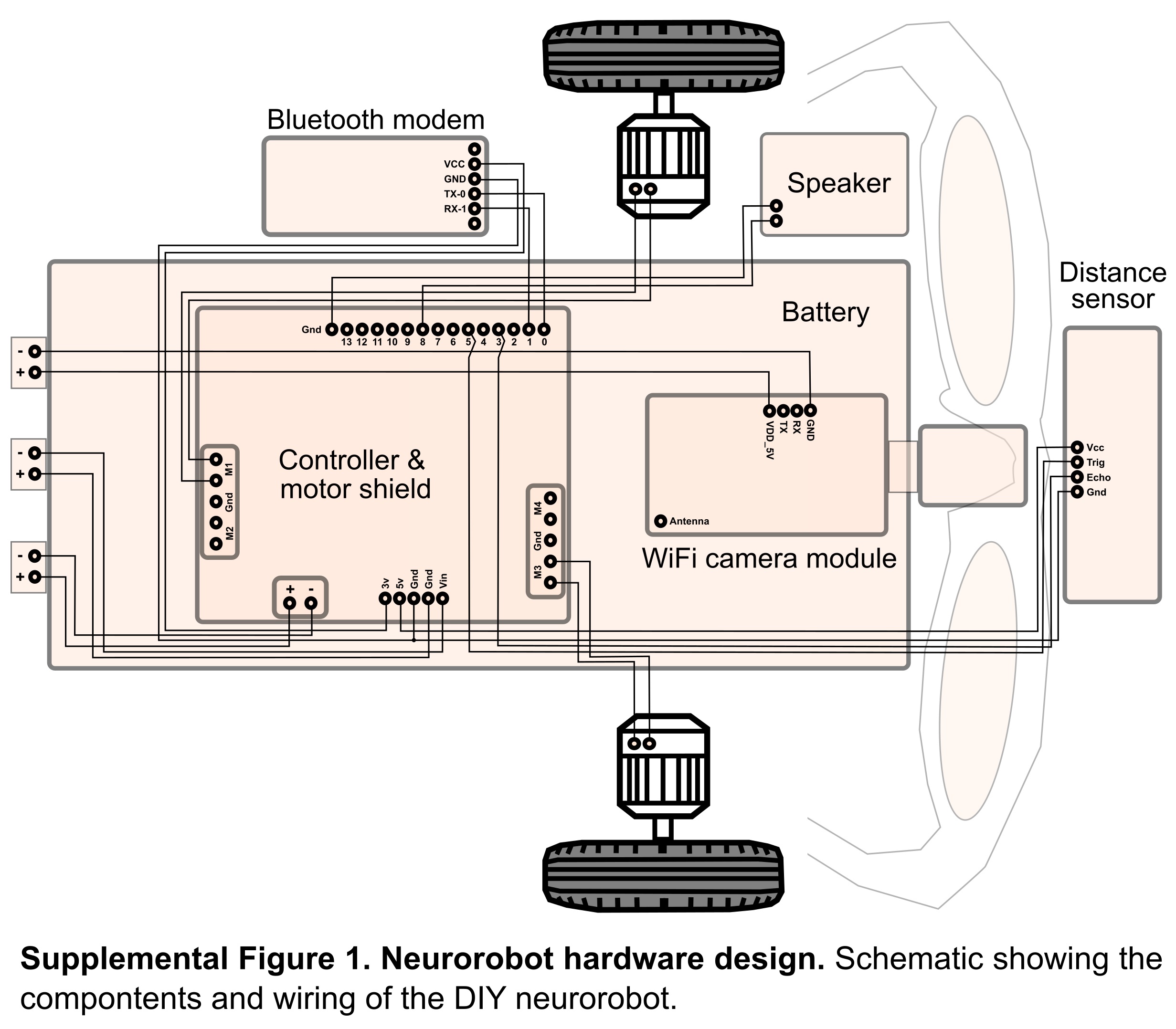

Supplement: Supplementary file 2 [file Image_1.jpg]
